# Sequential combination of decitabine and idarubicin synergistically enhances anti-leukemia effect followed by demethylating Wnt pathway inhibitor promoters and downregulating Wnt pathway nuclear target

**DOI:** 10.1186/1479-5876-12-167

**Published:** 2014-06-12

**Authors:** Kongfei Li, Chao Hu, Chen Mei, Zhigang Ren, Juan Carlos Vera, Zhengping Zhuang, Jie Jin, Hongyan Tong

**Affiliations:** 1 Department of Hematology, the First Affiliated Hospital of Zhejiang University, Hangzhou 310003, Zhejiang Province, People’s Republic of China; 2 Department of Hematology, Yin Zhou People Hospital, Ningbo 315040, Zhejiang Province, People’s Republic of China; 3 Institute of Hematology, Zhejiang University School of Medicine, Hangzhou 310009, Zhejiang Province, People’s Republic of China; 4 Department of Hepatobiliary and Pancreatic Surgery, the First Affiliated Hospital of Zhejiang University, Hangzhou 310003, Zhejiang Province, People’s Republic of China; 5 Surgical Neurology Branch, National Institute of Neurological Disorders and Stroke, National Institutes of Health, Building 10, Room 5D37, 9000, Rockvillle Pike, Bethesda, MD 20892, USA; 6 Department of Hematology, Sir Run Run Shaw Hospital of Zhejiang University, Hangzhou 310016, Zhejiang Province, People’s Republic of China

**Keywords:** Decitabine, Idarubicin, Wnt, Acute myeloid leukemia, Myelodysplastic syndromes

## Abstract

**Background:**

The methylation inhibitor 5-Aza-2′-deoxycytidine (decitabine, DAC) has a great therapeutic value for acute myeloid leukemia (AML) and myelodysplastic syndromes (MDS). But decitabine monotherapy was associated with a relatively low rate of complete remission in AML and MDS. We aimed to investigate the effect of several anti-leukemia drugs in combination with decitabine on the proliferation of myeloid leukemia cells, to select the most efficient combination group and explore the associated mechanisms of these combination therapies.

**Methods:**

Cell proliferation was tested by MTT assay and CFU-GM assay. Cell apoptosis was evaluated by Annexin V and PI staining in cell culture, TUNEL assay and transmission electron microscopy in animal study. MicroPET was used to imaging the tumor in mouse model. Molecular studies were conducted using microarray expression analysis, which was used to explore associated pathways, and real-time quantitative reverse transcription-PCR, western blot and immunohistochemistry, used to assess regulation of Wnt/β-catenin pathway. Statistical significance among groups was determined by one-way ANOVA analysis followed by post hoc Bonferroni’s multiple comparison test.

**Results:**

Among five anti-leukemia agents in combining with decitabine, the sequential combination of decitabine and idarubicin induced synergistic cell death in U937 cells, and this effect was verified in HEL, SKM-1 cells and AML cells isolated from AML patients. Importantly, tumor growth inhibition in this sequential combination was found to be higher than in single agent or controls in vivo. Moreover, sequential combination of the two agents induced apoptosis and depression of the Wnt/β-catenin pathway in both AML cell culture and animal studies.

**Conclusions:**

The findings demonstrated that sequentially combination of decitabine and idarubicin had synergistic anti-leukemia effects. These effects were mainly attributed to demethylation of Wnt/β-catenin pathway inhibitors and downregulation of Wnt/β-catenin pathway nuclear targets.

## Introduction

5-Aza-2′-deoxycytidine (decitabine, DAC), an analog of deoxycytidine, contains a nitrogen group substituted for C-5 of the pyrimidine ring [1]. DNA polymerase facilitates the insertion of DAC into DNA during the replication step of transcription, which upon occurring, leads to a permanent combination with DNA methyltransferase (DNMT). By binding DNMT, DAC lowers the enzyme’s expression levels and bioactivity and causes demethylation of hypermethylated DNA, which induces re-expression of silenced genes [2,3]. As previously reported, low doses of DAC induce epigenetic modulation, while high doses have cytotoxic effects [4]. Given the association between DAC-mediated hypomethylation and reactivation of multiple genes, some groups have looked to this drug for its important role in the control of cell proliferation and differentiation [5].

In practice, DAC has been an effective therapy for acute myeloid leukemia (AML) and myelodysplastic syndromes (MDS). Recently, DAC monotherapy was associated with a relatively low rate of complete remission rates in AML and MDS [6-8]. Kantarjianet al. reported in a phase III randomized study of DAC in treatment of 170 MDS patients, the overall response rate (OR) was 17%, including 9% complete responses [7]. Furthermore, Issa et al. conducted a Phase I study of 37 patients with AML receiving DAC in which the OR was 17% [8].

Several groups have attempted to increase the response rate of DAC-based therapy by developing combinations treatments [9,10]. By and large, these have taken on three varieties: combining DAC with other epigenetic modulating agents, cytotoxic agents, and using DAC as a biologic response modifier to increase the efficacy of other drugs. Since the effects of these combined therapies are not ideal, it is necessary to explore novel combinations. In this study, we have investigated the effect of five anti-leukemia drugs (idarubicin, IDA; daunorubicin, DNA; aclarubicin, ACLA; thalidomide, THAL; and homoharringtonine, HHT) in combination with DAC, given either simultaneously or sequentially, on proliferation in various AML cell lines.

## Materials and methods

### Reagents

DAC was supplied and formulated by Pharmachemie BV, Haarlem, the Netherlands. HHT was purchased from Minsheng Pharmacia (Zhejiang, China). IDA and DNR were purchased from Haizheng Pharmacia (Zhejiang, China). ACLA was purchased from Wanle Pharmacia (Shenzhen, China). THAL was purchased from Sigma (St. Louis, MO, USA). DAC was used immediately after dissolving it in phosphate buffer saline (PBS). Other agents were dissolved in PBS and stored at -40°C.

### AML samples

Bone marrow samples were collected during routine diagnostic assessment after written informed consent had been obtained. Patient disease was characterized using FAB classification, leading to grouping of patient 1 and patient 3 in AML-M_5_ category with more than 90% blast cells and patient 2 into AML-M_2_ category with 80% blast cells; three healthy volunteers were selected as normal controls. Patients’ mononuclear cells were separated by Ficoll-Hypaque (Sigma Chemical Co.) density-gradient centrifugation and used immediately. All participants provided written informed consent prior to entering the study. The study conformed to the ethical guidelines of the 1975 Declaration of Helsinki and was approved by the Institutional Review Board of the First Affiliated Hospital of Zhejiang University.

### Cell culture

Human AML cell lines, U937 promonocytic human cell line [11] and HEL erythroleukemia human cell line [12], were obtained from the Shanghai Cell Culture Institute (Shanghai, China). SKM-1 was established from a patient with progression to myelomonocytic leukemia in MDS, which was defined as refractory leukemia cell line [13]. It was obtained from the Health Science Research Resources Bank (Osaka, Japan). The cells were grown in RPMI 1640 plus 10% GIBCO FCS in plastic tissue culture plates in a humidified atmosphere containing 5% CO_2_ at 37°C. For the growth inhibition assay, the leukemic cell lines were cultured at a density of 2 × 10^5^ cells/mL and mononuclear AML cells were cultured at 5 × 10^5^ cells /mL in the medium before treatment.

### Cell proliferation assay

Cell proliferation was tested by colorimetric 3-(4, 5-dimethylthiazol-2-yl)-2, 5-diphenyltetrazollium bromide (MTT) assay. MTT (Sigma, USA) was dissolved in PBS at 5 mg/mL and used to measure cell viability. Briefly, aliquots (200 μl) of the cell suspension were dispensed into 96-well flat-bottomed microplates containing various chemical dilutions in six replicate rows. The plates were incubated in a humidified incubator in 5% CO_2_ for 72 h at 37°C with 20 μl of MTT solution (5 mg/mL MTT in phosphate-buffered saline (PBS) stored at 4°C). After the resulting solution was incubated in 5% CO_2_ for another 4 h at 37°C, Formazan crystals were dissolved in 200 μl of DMSO. The plates were then analyzed on an enzyme-linked immunosorbent assay plate reader at 570 nm. All experiments were performed in triplicate.

### Colony forming unit granulocyte/macrophage (CFU-GM) assay

AML cell lines (U937, HEL and SKM-1) were cultured at different concentrations (5 × 10^3^/mL) in Iscove’s modified Dulbecco’s medium (IMDM) (GIBCO-Life Technologies, Paisley, UK) containing 20% fetal bovine serum (FBS) (Hyclone, Logan, UT, USA), 0.3% agar (Difco, Detroit, MI, USA) and variable concentrations of either human recombinant GM-CSF (Myelogen, from Schering-Plough, Milan, Italy) or human recombinant IL-3 (gift from Sandoz, Basel, Switzerland). Negative and positive control cultures were also established, with recombinant growth factors replaced by IMDM and 10% supernatant of U937, HEL and SKM-1, respectively. Colonies with at least 50 cells were scored after 14 days of culture.

### Apoptosis detection by Annexin V and PI staining

Cells were treated with fresh drug preparations and medium daily for 3 days. Cells were then washed in PBS and resuspended in 100 μl of binding buffer [10 mm Hepes/NaOH (pH 7.4), 140 mm NaCl, and 2.5 mm CaCl_2_] containing Annexin V 5 μl (BD Pharmingen, SanDiego, CA, USA). The cells were analyzed by flow cytometry after the addition of 5 μl propidium iodide (PI). Annexin V binds to cells that express phosphatidylserine on the outer layer of the cell membrane, while PI stains the cellular DNA of those cells with a compromised cell membrane. This allows for viable cells (unstained with either fluorochrome) to be distinguished from apoptotic cells (stained only with Annexin V) and necrotic cells (stained with both Annexin V and PI).

### Methylation-specific polymerase chain reaction

Genomic DNA was prepared from cells and subsequent bisulfite conversion of genomic DNA was carried out. The methylation status of the CpG islands in the SFRP1, DKK3 and HDPR1 gene promoters were determined by methylation-specific PCR (MSP) as described by Griffiths EA et al. [14]. DNA was amplified according to the following protocol: 95°C for 5 min, followed by 40 cycles of 95°C for 1 min, 60°C for 30s, 72°C for 1 min, and a final extension step of 72°C for 10 min. Amplified products were resolved on 3% agarose gels and visualized under ultraviolet light after staining with ethidium bromide. DNA from normal donors was used as negative control. Human cell-line DNA treated in vitro with SssI methylase (New England Biolabs) was used as a positive control. Results were confirmed by repeat MSP assays after an independently performed bisulfite treatment.

### Real-time quantitative reverse transcription-PCR

Total cellular RNA was isolated from cells using Trizol (Gibco-BRL). RNA was eluted with RNase-free water, quantified at an absorbance at 260/280 nm, and used for reverse transcription reaction. The primer sequences are present in Table 1. Real-time quantitative reverse transcription-PCR was performed in an optional 96-well plate with Bio-Rad iQ5 system (Bio-Rad, USA) and a commercial SYBR-Green master mix kit (Takara, Japan), according to the manufacturer’s instruction. Assembled plates were then covered and run using the following conditions: an initial denaturation step of 95°C for 10 min followed by 45 cycles at 95°C for 15 s and 60°C for 1 min. Each sample was measured in triplicate. The resulting data were analyzed with Bio-Rad iQ5 Optical System Software version 2.0 and shown as the mean ± SD of three determinations for each sample.

**Table 1:** The primer sequences used in Real-time quantitative reverse transcription-PCR

***Gene name***	***Sequences***		***Length***
	***F(5′—3′)***	***R(3′—5′)***	
SKRP-1	ACTGGCCCGAGATGCTTAAGTG	GAGATGTTCAATGATGGCCTCAGA	158 bp
DKK3	TGCTGCTAAAGCATCATCAGAAGTG	ACCATTTGTCCAGTCTGGTTGTTG	150 bp
HDPR1	TGAGCATCCGGCAAGGTACA	CATGGAGACACTGCCCTGAAGA	131 bp
β-catenin	GAGTGCTGAAGGTGCTATCTGTCTG	TTCTGAACAAGACGTTGACTTGGA	115 bp
C-MYC	CCTGGTGCTCCATGAGGAGA	TCCAGCAGAAGGTGATCCAGAC	145 bp
Cyclin D1	ATGTTCGTGGCCTCTAAGATGA	CAGGTTCCACTTGAGCTTGTTC	138 bp

### Western blotting

Cultured cells were collected in lysis buffer (Cell Signal Technology, Boston, MA, USA). Equal amounts of proteins were separated on 10% sodium dodecyl sulfate-polyacrylamide gel electrophoresis (SDS-PAGE) and transferred onto PVDF membranes (Millipore, Boston, MA, USA). Membranes were blocked with 5% skim milk, incubated with primary antibodies at 4°C overnight in TBS-T (10 mm Tris–HCl, pH 8, 150 mm NaCl, 0.1% Tween 20). The primary antibodies used were as follows: β-actin, β-catenin, c-Myc, CyclinD1, SFRP1 (Cell Signal Technology), DKK3 and HDPR1 (Abcam, Cambridge, MA). After incubation with peroxidase-conjugated secondary antibodies for 2 hours, blots were revealed by enhanced chemiluminescence procedures according to the manufacturer’s recommendation.

### Microarray expression analysis

The cells with different treatment were collected for total RNA extraction using Trizol reagent (Invitrogen, CA, USA) and purification using RNeasy Mini Kit (QIAGEN, 74106) following the manufacturer’s instructions. Microarray expression assays were performed at the ShanghaiBio Corporation (National Engineering Center for Biochip in Shanghai, China) using the Agilent 4x44K microarrays. The raw data were normalized using quantile normalization and then analyzed in GeneSpring GX (zcomSilicon Genetics, Redwood City, CA, USA). Differentially expressed genes showing a statistical significance (*p* < 0.05, one way analysis of variance) and ≧3-fold change between each treatment groups were further analyzed using SBC analysis system (Biochip, Shanghai, China): Gene Ontology (GO) analysis was applied to organize genes into hierarchical categories on the basis of cellular component, biological process and molecular function; enrichment pathway analysis (Kegg database) was implemented to uncover the major perturbed pathways. The microarray gene expression data have been submitted in NCBI’s Gene Expression Omnibus (GEO, http://www.ncbi.nlm.nih.gov/geo/) under accession number GSE54918.

### Subcutaneous AML model of NOD/SCID mouse

NOD/SCID mice were purchased from Shanghai laboratory animal center. AML cell line, U937 (1×10^7^ cells per animal), was injected subcutaneously into the right flank of 6 to 8-week old NOD-SCID mice. When tumors reached a volume of ~50 mm^3^, animals were randomly assigned to one of four groups. The total number of mice was 16, divided into four groups with 4 each. Either DAC (0.5 mg/kg/day) for five consecutive days followed by a three days of IDA (0.5 mg/kg/day) or DAC for five days alone and IDA for three days alone. The untreated control received PBS. Drugs and PBS were injected intraperitoneally. Tumor volume was measured and calculated as π/6 length × width2. On Day 7 after treatment, tumors were evaluated by micro PET. One mouse from each group was killed according to ethical animal practice, and tumors were harvested to determine cell apoptosis expression levels of the Wnt/β-catenin pathway. All procedures were performed in accordance with the “Guide for the Care and Use of Laboratory Animals” published by the National Institutes of Health (NIH publication 86–23 revised 1985). Animal protocols were approved by Animal Care and Facilities Committee of Zhejiang University.

### Immunohistochemical staining

Paraffin-embedded tumor tissues from mice were sectioned and deparaffinized with xylene. The slides were immersed into different concentrations of alcohol (100%, 95%, 75%, 50%, ddH_2_O) for rehydration and then in 3% H_2_O_2_ to block endogenous peroxidase. For antigen retrieval, slides were immersed in boiling (95°C -100°C) citrate buffer (pH 6.0) for 20 min. After washing with PBS, the slides were soaked in blocking solution (3% BSA) at room temperature for 30 min. The slides were incubated with primary antibody against β-catenin (Cell Signaling, Beverly, MA) at 4°C overnight. After rinsing with PBS, the secondary antibody, HRP Polymer Conjugate Reagent (SuperPicture Polymer Detection kit), was added for 10 min and DAB Chromogen for 5 min. At each incubation step, slides were followed with washing in PBS for 5 min. Mayer’s Hematoxylin solution was used for counterstaining. Finally, slides were dehydrated, air-dried and mounted. For hematoxylin and eosin staining (H&E stain) sectioned slides were placed in hematoxylin solution for 15 min, washed with ddH_2_O and then counterstained with eosin for 5 min.

### Measurement of apoptotic cells with TUNEL assay

Cell apoptosis was measured using a TdT–dUTP Terminal Nick-end Labeling (TUNEL) assay and In Situ Cell Death Detection Kit (Millipore, USA), according to the manufacturer’s protocol. Briefly, Paraffin-embedded tumor tissues from mice were sectioned and deparaffinized with xylene. The slides were immersed into different concentrations of alcohol (100%, 95%, 75%, 50%, ddH_2_O) for rehydration and then in 3% H_2_O_2_ to block endogenous peroxidase. The slides were then washed using PBS and incubated for 5 min on ice with permeabilization solution (0.1% triton X-100 in 0.1% sodium citrate). The cells were then treated using TUNEL response mixture [450 ml of label solution (fluorescein–dUTP) and 50 ml of enzyme solution (TdT)] for 1 h at 37°C in a humid environment. The label was then incorporated into damaged sites of DNA. The apoptotic cell was stained brownish–yellow and observed under light microscopy. TUNEL positive cells were analyzed with the Image Pro-Plus software.

### Transmission electron microscopy

Tumor tissue was prepared for transmission electron microscopy (TEM) by the standard technical procedures. Tumor ultrastructure was observed by TEM in Imaging Facility of Core Facilities, Zhejiang University School of Medicine, as previously described [15]. Briefly, tumor sample was fixed in 2.5% glutaraldehyde (4°C, pH 7.4), postfixed in 1% osmium tetroxide, and embedded in an epon–araldite mixture. Ultrathin sections of the tumor tissue were obtained and placed onto mesh copper grids and stained with uranyl acetate and lead citrate. The cell microstructure was observed on a Philips Tecnai 10 electron microscope (Philips, Eindhoven, Netherlands). Cell morphology especially the nuclear and mitochondria were observed.

### MicroPET

An R4 micro PET (CTI Concorde Microsystems, LLC) was used for imaging, and for each mouse in the study, we acquired 3-D FDG-micro PET images. After a tail vein injection of 3.7–7.4 MBq of 18F-FDG in 200 mL of PBS, a 7-min prone acquisition scan was performed approximately 60 min after injection. Mice were maintained under isoflurane anesthesia during the injection, accumulation, and scanning periods. A heating pad, heat lamp, or hot water was used to dilate the tail veins for injections. The standardized uptake value (SUV) was calculated by ASIPro 6.0.5.0 software.

### Statistical analysis

Statistical significance among groups was determined by one-way ANOVA analysis followed by post hoc Bonferroni’s multiple comparison test. For non-parametric data, Kruskal-Wallis followed by Dunn’s multiple comparison test was used. P values less than 0.05 were considered statistically significant. All data were presented as mean ± standard deviation (SD). GraphPad PrismV5.0 (GraphPad Software, San Diego, CA) was used for statistical analysis. * *p* < 0.05, ** *p* < 0.01, ***p < 0.001.

## Results

### Selection of effective drugs combined with DAC in AML cell line U937

Individual anti-leukemia drugs (IDA, DNR, ACLA, THAL, and HHT) were combined with DAC to investigate the effect of inhibiting proliferation of AML cells. Three different ways of combining DAC and anti-leukemia drugs were studied (see Additional file 1: Table S1): simultaneous combination (group 1) and sequential combination, separated into two groups (group 2 and group 3) depending on number of times DAC was given. Drug dosages were selected after calculating IC_50_ (see Additional file 1: Table S1) using the median-effect method. For the simultaneous combination group (group 1), U937 cells were treated with different concentrations of DAC and anti-leukemia drugs concurrently. In sequential combination groups, DAC was given to the U937 only once at the first day (group 2) or once a day for the first two days (group 3), and anti-leukemia drug was added at the third day. In all combination groups, the timing for the IC50 of DAC was 72 h. The timing for the IC50 of the other drugs (HHT, THAL, ACR, DNR, and IDA) was 72 h in combination group 1, 24 h in group 2 and 3.

**Figure 1:**
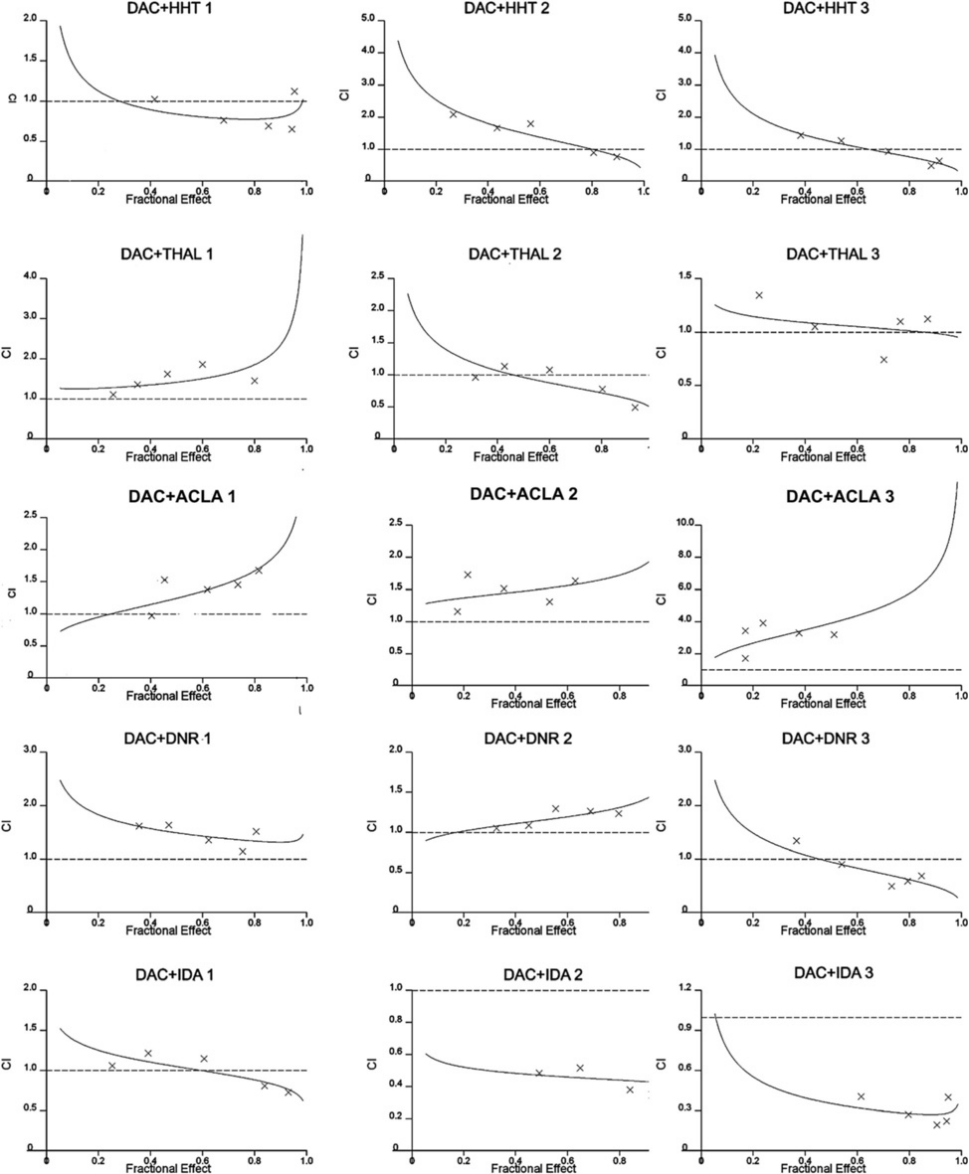
**CI plots of DAC and anti-leukemia drugs combined effect in human myeloid leukemia cell line U937.** The effect of the combinations was assessed by MTT after 3 day’s culture with drugs. Cells were divided into three groups. The first column, cells treated with DAC and anti-leukemia drugs simultaneously at the first day. The second column: DAC was added at the first day, anti-leukemia drugs at the third day. The thirst column: DAC was added at the first and second day, and anti-leukemia drugs at the third day. The effects of combinations were estimated using the CalcuSyn software, which was developed based on the median-effect method. CI < 0.8 indicates synergy; CI = 0.8 to 1.2 is additive; and CI > 1.2 means antagonism. The result showed that DAC sequentially combined with IDA had most notable synergistic effect among the five anti-leukemia drugs simultaneously or sequentially combining with DAC, and sequential combination with twice DAC given were more effective than the once DAC given when DAC combined with IDA.

The effects of combinations were estimated using the CalcuSyn software, which was developed based on the median-effect method created by Chou et al. [16]. Representative experiment was generated from at least 3 independent experiments for each cell line. The CI50 were reported as mean ± SD (see Additional file 2: Table S3). After combining DAC with HHT, ACLA, THAL and DNR both simultaneously and sequentially, the CI values at the various doses were almost over 0.8. This meant that no synergistic effect occurred in the combinations. Moreover, no synergistic effect was also observed in the simultaneous combination of DAC and IDA. But when DAC was sequentially combined with IDA, its CI values on all five doses were under 0.8. The CI values of the sequential combination with twice given DAC were lower than the once given DAC (Figure 1). This result showed that DAC sequentially combined with IDA had the most notable synergistic effect among the five anti-leukemia drugs simultaneously or sequentially combined with DAC.

### Synergistic effect of sequential combining DAC and IDA in inhibiting AML cell proliferation

**Figure 2:**
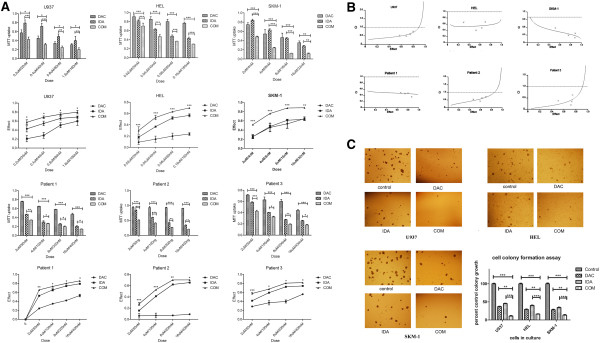
**Synergistic effect of sequential combining DAC and IDA in inhibiting AML cell proliferation. A**, Effects of DAC sequentially combining with IDA on cell growth in leukemia cell lines. Cells were treated with DAC twice given for 2 days, and then treated with IDA for 1 day. Cell viability was measured by MTT and IC50 was calculated by the median-effect method. The MTT uptake figures and the effect figures were analyzed by PrismV5.0 (GraphPad Software, San Diego, CA). Statistical differences were calculated by one-way ANOVA. *p < 0.05, **p < 0.01, ***p < 0.001. **B**, CI plots of DAC and IDA sequential combination in human leukemia cell lines. Cells were treated as previously described. The combinations were present in a fixed molar ratios based on the IC50 values of each drug. The effects of combinations were estimated using the CalcuSyn software, which was developed based on the median-effect method. CI < 0.8 indicates synergy; CI = 0.8 to 1.2 is additive; and CI > 1.2 means antagonism. **C**, AML cell lines U937, HEL and SKM-1 were treated with DAC, IDA, or DAC combined with IDA, respectively, and the survival of AML cells was evaluated by colony formation assays. Mean ± SD. ***p < 0.001, **p < 0.01, *p < 0.05.

To evaluate the effects of sequentially combining DAC and IDA on AML cell viability, we treated U937 cells, other AML cell lines (HEL, SKM-1) and cells from AML patients using different drug concentrations according to their IC50 of each compounds (see Additional file 3: Table S2). The timing for IC50 of DAC was 72 h for U937, HEL and cells from patients, 96 h for SKM-1. The timing for IC50 of IDA was 24 h for the three cell lines and cells from patients. U937 cells were treated with DAC: 0.2, 0.4, 0.8 and 1.6 μmol/L for 2 days, and sequentially with different concentrations of IDA: 20, 40, 80 and 160 nmol/L for 1 day; HEL cells with DAC: 0.02, 0.04, 0.08 and 0.16 μmol/L for 2 days, IDA: 20, 40, 80 and 160 nmol/L for 1 day; SKM-1 cells with DAC: 2, 4, 8 and 16 μmol/L for 3 days; IDA: 4, 8, 16 and 32 nmol/L for 1 day; AML cells from Patients with DAC: 2, 4, 8 and 16 μmol/L for 2 days; IDA: 50, 100, 200 and 400 nmol/L for 1 day. Since SKM-1 was established from a patient with progression to myelomonocytic leukemia in myelodysplastic syndrome (MDS), which was defined as refractory leukemia [15], it was considered less sensitive to DAC than other AML cell lines U937 and HEL. So we treated SKM-1 cells with DAC for one more day than other cells. Percentages of live/viable cells in treated plates were measured using MTT proliferation assay and data collected was analyzed by GraphPad Prism.V5.0 software (Figure 2A). The effects of combinations were estimated using the CalcuSyn software. Representative experiment was carried out from at least 3 independent experiments for each cell line, and at least twice for cells from AML patients. The CI50 were reported as mean ± SD (see Additional file 2: Table S3). The sequential combination of DAC and IDA were synergistic in all studied AML cells, indicated by combination index (CI) values of <0.8 (Figure 2B). The CI value in U937 cells treated with DAC followed by IDA ranged from 0.53 to 0.76 (in Hel: 0.40-0.67; in SKM-1:0.19-0.57; patient 1: 0.35-0.52; Patient 2: 0.42-0.71; Patient 3: 0.29-0.79), indicating inhibited proliferation of AML cells achievable by adding DAC before IDA. CFU-GM assays were done to assess the effect of sequentially combining DAC and IDA on AML cells by plating appropriate numbers of AML cells in methylcellulose medium. The data (Figure 2C) revealed that the number of AML cell clones was significantly affected when combining DAC and IDA sequentially, as compared to treatment groups using DAC or IDA alone. These results indicate that the sequential combination of DAC and IDA has a synergistic effect on the AML cell proliferation in vitro.

### Sequential combination of DAC and IDA efficiently inhibits tumor growth in subcutaneous AML mouse model

**Figure 3:**
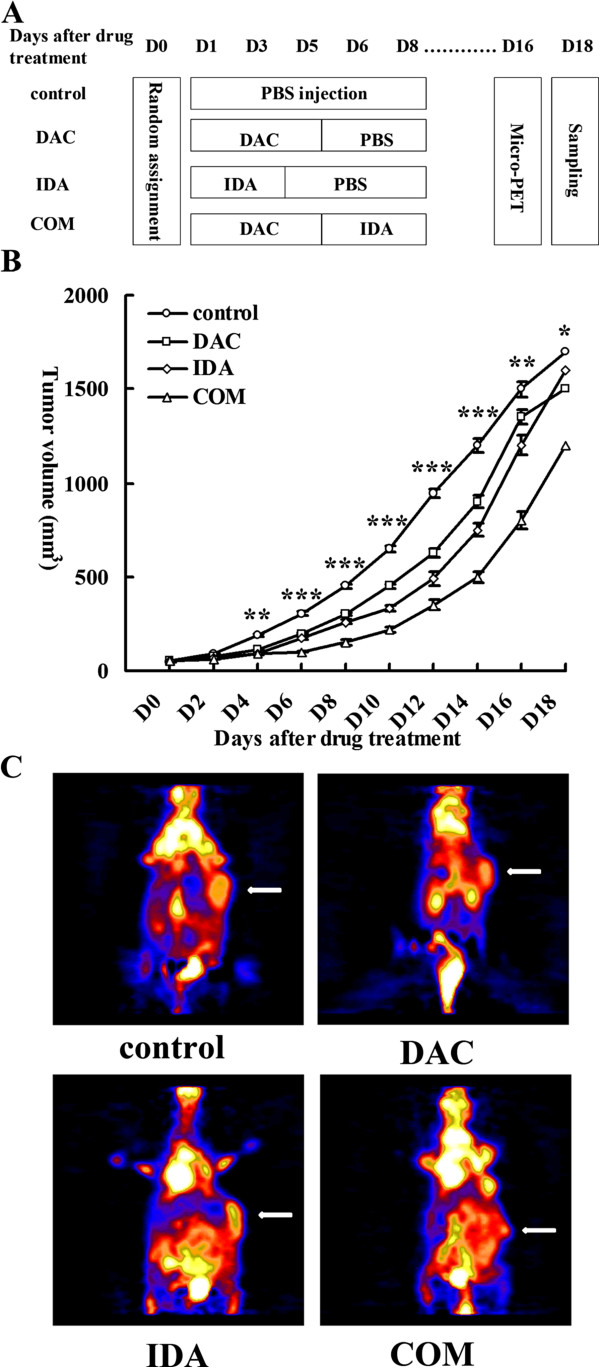
**Sequential combination of DAC and IDA efficiently inhibits tumor growth in subcutaneous AML mouse model. A**, Outlines of the animal experiment protocol. **B**, Effect of DAC sequentially combining with IDA on tumor volume in a subcutaneous AML mouse model transplanted with U937 cells. Tumor volume is expressed as Mean ± SD. (n = 4). Statistical differences between groups were analyzed with one-way ANOVA, and statistical significance was defined as *p* < 0.05. (*) indicates statistically significant differences between the combined group and the control group. **C**, 18F-FDG micro PET image of subcutaneous AML mouse model treated with FBS, DAC alone, IDA alone and DAC followed by IDA. The white arrows point to the area of the subcutaneous AML tumor. The SUV of control group was 8.25, DAC group was 6.32, IDA group was 7.09, and DAC combining with IDA group was 4.77.

In vivo, we designed the control group strictly (Figure 3A), and observed tumor growth dynamically. Tumor growth in the combined group versus control group, presented by tumor growth curve (Figure 3B, mean ± SD), was inhibited on the day 4 after drug treatment (91 ± 1.9 vs. 189 ± 3.0 mm3, p < 0.01), significantly inhibited on the day 6 (100 ± 2.0 vs. 305 ± 5.9 mm3, p < 0.001), day 8 (150 ± 3.6 vs. 450 ± 8.4 mm3, p < 0.001), day 10 (219 ± 15 vs. 650 ± 10 mm3, p < 0.001), day 12 (350 ± 14 vs. 945 ± 15 mm3, p < 0.001), day 14 (500 ± 25 vs. 1200 ± 22 mm3, p < 0.001), and day 16 (800 ± 29 vs. 1500 ± 35 mm3, p < 0.01) after drug treatment. On the day 18 after drug treatment, the inhibition effect become decreased (1200 ± 46 vs. 1700 ± 40 mm3, p < 0.05), along with the lengthened time after drug administration. Meanwhile, the significant inhibition of tumor growth was noted through the tumor imaging by micro-PET on the day 16 after drug treatment (Figure 3C).

### Synergism of sequential combining decitabine and idarubicin in inducing apoptosis of AML cells and tumor cells of subcutaneous AML mouse model

**Figure 4:**
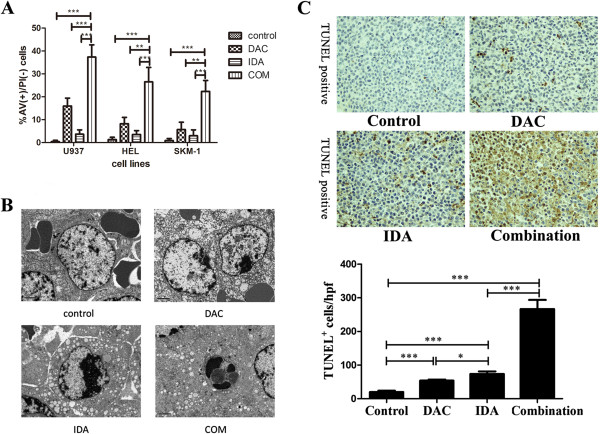
**Synergism of sequential combining decitabine and idarubicin in inducing apoptosis of AML cells and tumor cells of subcutaneous AML mouse model. A**, Annexin V and PI staining for apoptosis assay in U937, HEL and SKM-1 cells. Apoptosis was assayed by Annexin V and PI staining and fluorescence-activated cell sorting analysis. The percentage of apoptotic cells (Annexin V and PI positive) was indicated in the histogram. **B**, The Morphological and ultrastructural changes in apoptosis cells. To compare with the control group, DAC group and IDA group, the tumor cells from subcutaneous AML mouse model treated with DAC followed by IDA some showed the typical characteristic of apoptosis cells which microvilli on cell membrane were disappeared, bubbles exist on nuclear and cell membrane, chromosome condensation and the formation of apoptotic bodies. **C**, TUNEL assay of tumor tissue from subcutaneous AML mouse model confirmed induction of apoptosis after treatment with DAC alone, IDA alone and DAC followed by IDA. ***p < 0.001, **p < 0.01, *p < 0.05.

To determine whether apoptotic cell death is responsible for DAC and IDA induced decrease in cell viability, we performed flow cytometry analysis with Annexin V and PI staining. AML cells were treated with different concentrations: U937 with DAC 0.8 nmol/L for 48 h followed by IDA 40 nmol/L for 24 h, HEL with DAC 0.08 nmol/L for 48 h followed by IDA 40 nmol/L for 24 h, SKM-1 with DAC 8 μmol/L for 72 h followed by IDA 8 nmol/L for 24 h, according to the concentrations of each compounds used in the inhibition of AML cells proliferation. The combinatory effect of DAC and IDA on AML cell apoptosis was observed, and the results showed that the apoptosis rates (Annexin V + and PI-) in the combined group were significantly increased in the U937, HEL and SKM-1 cell lines compared with the control, DAC and IDA groups (all p < 0.001, Figure 4A). Meanwhile, we determined the morphological and ultrastructural changes in tumor tissue from subcutaneous AML mouse model by TEM. Compared with the control, DAC and IDA groups, the tumor cells treated with DAC followed by IDA showed the obvious apoptosis characteristics: absence of microvilli on cell membrane, nuclear and cell membrane blebbing, chromosome condensation, and the formation of apoptotic bodies (Figure 4B). We also performed the TUNEL assays to confirm the apoptosis effect of DAC and IDA sequential combination treatment, and the staining results demonstrated that the TUNEL-positive cells/hpf in tumor tissue were remarkably increased in the combination group versus the other groups (all p < 0.001) (Figure 4C). These results suggested that the combinational interaction between the two drugs significantly inhibited tumor growth mainly through inducing tumor cells apoptosis.

### Disrupted gene expression profile by sequential combination of DAC and IDA in AML cells

To gain insights into the mechanism of the combination effect of DAC and IDA, we used microarray expression assay to find the differential expression genes between combination and other three treatment groups (combination vs. no-treatment control, combination vs. DAC, combination vs. IDA) (raw data not shown). In order to find the most sensitive genes to the drugs, we chose a lower dose than IC50 of each drug. U937 cells were treated with PBS for 72 h, DAC 0.2 μmol/L for 72 h with once a day given at the first two days, IDA 20 nmol/L for 24 h, and DAC 0.2 μmol/L for 48 h with once a day given at the first two days followed by IDA 20 nmol/L for 24 h. A total of 2294 genes were found to be differentially expressed by at least three-fold between the combination group and no-treatment control group, 643 genes between the combination and DAC groups, 1655 genes between the combination and IDA groups. The differently genes were processed utilizing GO annotations and pathway analysis. In enrichment pathway analysis (Kegg), the differential expression genes between combination and other three groups are mainly involved in MAPK signaling pathway, TGF-beta signaling pathway and Wnt signaling pathway (Table 2). According to the P value and percent of differential gene expression between different treatment groups, Wnt pathway was one of the major perturbed pathways.

**Table 2:** The major perturbed pathways in combination comparing with other three groups respectively

***Groups***	***Pathways***	***Hits***	***Total***	***Percent***	***p value***
Combination vs. control (*p* < 0.001)	MAPK signaling pathway	42	271	15.5%	0.0
	TGF-beta signaling pathway	17	85	20.0%	0.0
	Natural killer cell mediated cytotoxicity	23	141	16.31%	0.0
	VEGF signaling pathway	9	76	11.84%	0.0147
	Wnt signaling pathway	18	151	11.92%	7.0E-4
Combination vs. DAC (*p* < 0.05)	MAPK signaling pathway	8	271	2.95%	0.0318
	p53 signaling pathway	3	69	4.35%	0.0689
	TGF-beta signaling pathway	4	85	4.71%	0.0298
	Wnt signaling pathway	9	151	5.96%	3.0E-4
Combination vs. IDA (*p* < 0.05)	MAPK signaling pathway	21	271	7.75%	8.0E-4
	Natural killer cell mediated cytotoxicity	19	141	13.48%	0.0
	p53 signaling pathway	9	69	13.04%	0.0011
	PPAR signaling pathway	7	69	10.14%	0.0128
	TGF-beta signaling pathway	12	85	14.12%	1.0E-4
	Wnt signaling pathway	16	151	10.6%	1.0E-4

### Methylation of Wnt antagonists and DNMTs expression in AML cells

**Figure 5:**
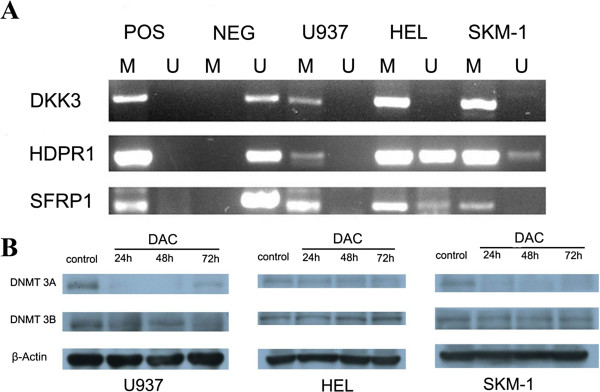
**Methylation of Wnt antagonists and DNMTs expression in AML cells. A**, MSP analysis. MSP was performed using previously published primer pairs as described in Methods. M, methylated reaction; U, unmethylated reaction; pos, positive control, neg, negative control. The PCR product for the M reaction was 158 bp long and that for the unmethylated reaction was 165 bp long; MSP, methylation-specific polymerase chain reaction. **B**, Expression of DNMT3A and DNMT3B in AML cell lines. Expression of DNMT3A and 3B were assayed by a standard western blot method in AML cells without treatment as the control group and AML cells with the treatment of DAC for 24 h, 48 h, and 72 h, respectively.

We used the methylation-specific PCR to examine the CpG methylation status of Wnt antagonists (DKK3, HDPR1, and SFRP1) in AML cell lines. Various degrees of hypermethylation of the gene promoters were observed in DKK3, HDPR1 and SFRP1 genes in U937, HEL and SKM-1 cell lines (Figure 5A). The results revealed complete methylation of DKK3 gene in all the three cell lines. The promoter of HDPR1 was also completely methylated in U937 cells but partial methylated in HEL and SKM-1 cells. SFRP1 was completely methylated in U937 and SKM-1 cells and partially methylated in HEL cell. Furthermore, we analyzed DNMT 3A and DNMT3B protein expression in AML cell lines after 24 h, 48 h and 72 h of 0.1 μmol/L DAC treatment. In U937 and SKM-1 cells, western blot analysis showed that DNMT3A expression was significantly repressed after treatment with DAC. Unlike in U937 and SKM-1 cells, DNMT3A expression was not significantly reduced after treatment with DAC in HEL cell line, which could be the results of cell-type specific effect of DAC. Nevertheless, DAC treatment did not significantly repress DNMT3B in any of the three AML cell lines tested (Figure 5B).

### Effect of sequential combination of DAC and IDA in inhibiting Wnt/β-catenin pathway in AML cells in vitro and tumor cells from a subcutaneous AML mouse model

**Figure 6:**
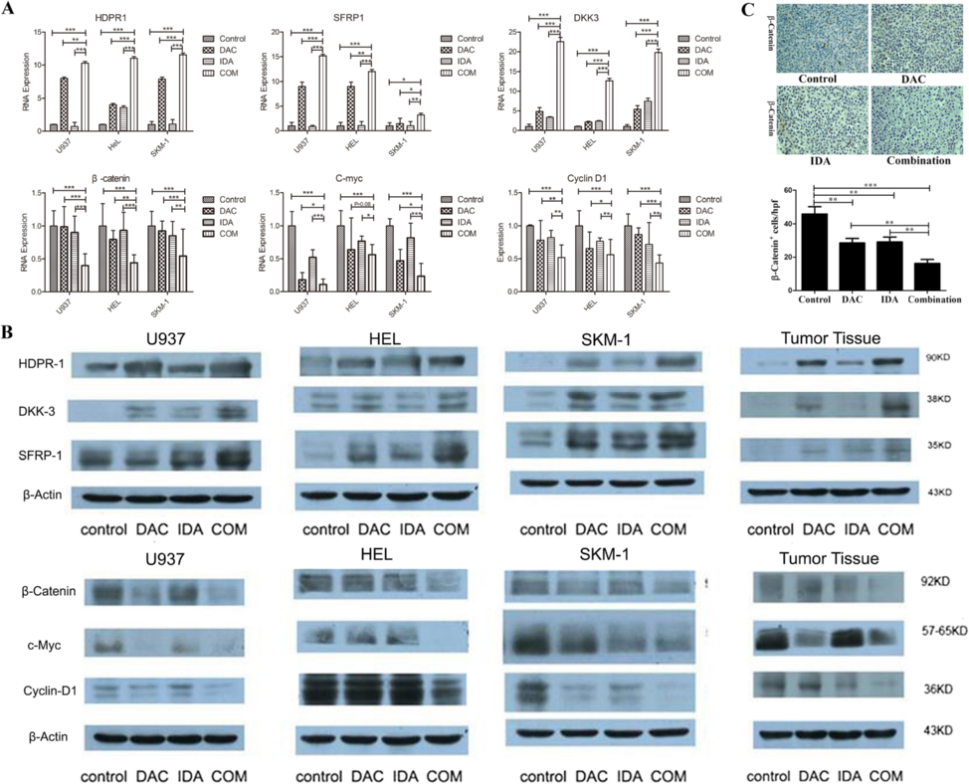
**Effect of sequential combination of DAC and IDA in inhibiting Wnt/β-catenin pathway in AML cells in vitro and tumor cells from a subcutaneous AML mouse model. A**, The mRNA expression of inhibitors and downstream genes of Wnt/β-catenin signaling pathway was measured by RT-PCR in control cultures, DAC treated cultures, IDA treated cultures and DAC followed by IDA treated cultures in the U937, HEL and SKM-1 cell lines. Expression was normalized in comparison with control cultures. The mean ± SD of four different experiments is shown. Statistical differences were calculated by one-way ANOVA. *p < 0.05, **p < 0.01, ***p < 0.001. **B**, Western blot analyze the protein expression of inhibitors and downstream genes of Wnt/β-catenin signaling pathway in four cultures of AML cell lines and tumor cells from AML mouse model. **C**, Immunohistochemistry for β-catenin in tumor tissues of subcutaneous AML mouse model. β-catenin expression was more frequently observed in group treated with DAC combining with IDA than treated with DAC or IDA alone.

RT-PCR and western blot were used to identify the DNA and protein extracted from the AML cells and tumor tissue. AML cells were treated with different concentrations: U937 with DAC 0.4 nmol/L for 48 h followed by IDA 40 nmol/L for 24 h, HEL with DAC 0.04 nmol/L for 48 h followed by IDA 40 nmol/L for 24 h, SKM-1 with DAC 4 μmol/L for 72 h followed by IDA 8 nmol/L for 24 h, according to the concentrations of each compounds used in the inhibition of AML cells proliferation. The xenograft mouse models were treated with DAC (0.5 mg/kg/day) for five consecutive days followed by a three days of IDA (0.5 mg/kg/day). And control groups were treated with single agent and PBS. Sequential treatment with DAC followed by IDA significantly up-regulated the Wnt antagonist genes (SFRP1, HDPR1 and DKK3) which resulted in re-expression or increased expression of these genes both at the mRNA and protein levels (Figure 6A, 6B). Furthermore, treating with IDA after DAC caused significantly down-regulation of the expression of c-Myc, β-catenin and CyclinD1 genes in AML cells, compared to treatments with DAC or IDA alone. We also found that the sequential treatment group induced re-expression or increased expression of the Wnt antagonist genes (SFRP1, HDPR1 and DKK3) in tumor cells of the subcutaneous AML mouse. The down-stream genes of Wnt/β-catenin pathway including β-catenin, c-Myc and CyclinD1 were also down-regulated, suggesting depression of the Wnt/β-catenin pathway. Western blot and IHC staining were used to detect expression of β-catenin in tumor cells of subcutaneously xenografted AML mice (Figure 6C).

## Discussion

DAC is a pyrimidine analogue used to inhibit DNMT. DNMT inhibition results in anti-leukemic activity are due to global and gene-specific DNA hypomethylation that induces re-expression of leukemia-related genes [17,18]. In the past, the investigators have shown that the subjects treated with DAC maintain normal hematopoietic stem cell self-renewal and display terminal differentiation of acute AML cells, hence its appeal in treating hematopoietic disorders like leukemia [19-21]. While the rates of complete remission using DAC are relatively low, however, hypomethylating-based combinations using DAC are being investigated to determine if further response rate improvement in myeloid malignancies can be achieved without burdening patients with adverse effects. To investigate the strategy, a number of hypomethylating combination trials for managing MDS and AML are underway and include the use of all-trans-retinoic acid (ATRA), arsenic trioxide (AS_2_O_3_), cytarabine (Ara-C) and topoisomerase I inhibitors [4,22-24]. Others are considering the role of combining histone deacetylase (HDAC) inhibitors and Lenalidomide [25,26]. Nevertheless, the most widely studied combination is that of DAC and HDAC inhibitors. This combination has been studied in phase I and early phase II trials in patients with AML and higher risk MDS, with reported OR ranging from 20 to 50% [9,10]. Willemze R et al. reported a randomized phase II study on the effects of DAC combined with IDA in patients with relapsed acute leukemia in 1993 and 1997, in which 15 of the 33 (45.5%) patients achieved a complete remission, higher than the combination of DAC and amsacrine. But no definite conclusions with respect to efficacy could be drawn as the patient population was quite small [27,28]. However, there has been no other evidence confirming whether DAC has a better effect when combined with the first-line anti-leukemia drugs, such as IDA and DNR. Exploring synergistic combinations and understanding the mechanisms underlying these pairings, especially in situations where DAC and other anti-leukemia drugs are paired, remains important.

In this study, we investigated five anti-leukemia drugs (IDA, DNR, ACLA, THAL, and HHT) in combination with DAC to observe their effect on the growth of myeloid leukemic cells. The results showed that DAC sequentially combined with IDA creates a synergistic effect of inhibiting the proliferation of AML cell lines, as well as cells from AML patients. Interestingly, the other anthracyclines including DNR and ACLA did not show a synergistic effect when combined with DAC. This suggests a specific biological mechanism resulting in a concerted effect. IDA is a synthetic analog of DNR and one of the most effective inhibitors of DNA topoisomerase. Alex et al. found a significant relation between apoptosis induction in leukemic cells by IDA in vitro and the disease remission measured by the percent of residual leukemic cells in bone marrow [29]. In this study, we found the sequential combination treatment of DAC and IDA efficiently inhibited the formation of AML cell clones and tumor growth in a subcutaneous AML mouse model. Furthermore, DAC sequentially combined with IDA presented markedly increased effect in inducing apoptosis of AML cells cultured in vitro and the tumor cells from the mouse model. We presume that the effect of DAC sequentially combined with IDA is mediated through apoptosis and inhibition of proliferation of cultured leukemic cells and tumor growth in a subcutaneous AML mouse model.

The temporal dependence of the combined treatments was also investigated, by testing the efficacy of DAC and IDA given simultaneously, DAC given once followed by IDA, and DAC given two or three times followed by IDA. We found that DAC added for two or more times followed by IDA resulted in the most synergistic activity when compared to the other treatment methods. A Phase I clinical trial of DAC prior to standard induction chemotherapy for patients with AML also demonstrated that demethylation of genes treated with DAC prior to chemotherapy might play a crucial role in enhancing the anti-leukemia effect [30], indicating that sequentially combining DAC with anti-leukemia drugs might be a better strategy for the design of a clinical trial. In our study, treating with DAC prior to IDA presented a synergistic effect in the AML cells. And this combination regimen significantly inhibited the tumor growth and induced apoptosis in a xenograft mouse model. These results suggested that DAC sequentially combined with IDA could enhance the anti-leukemia effect both in vitro and in vivo.

There are several molecular mechanisms and cell signaling pathways involved in tumor cell proliferation and apoptosis. In searching for the mechanism of synergy, we used microarray expression analysis to explore the associated pathways. Our results showed that the Wnt pathway was one of the most significantly perturbed pathways. It has been reported that the canonical Wnt pathway-Wnt/β-catenin signaling plays an important role in survival, proliferation and differentiation of hematopoietic stem cells, which ultimately contributes to the pathogenesis of leukemia [31-33]. The Wnt/β-catenin signaling pathway is an important target in several leukemogenic pathways supporting self-renewal, apoptosis-induction and proliferation of AML cells [34]. Different molecular mechanisms have been implicated in the abnormal activation of the Wnt/β-catenin signaling. Activation of Wnt/β-catenin signaling leads to inhibition of GSK-3β activity, resulting in accumulation of cytoplasmic β-catenin. This protein then becomes available to bind the TCF/LEF family of transcription factors and subsequently induces target proteins expression including c-Myc and cyclin D1 [33,35,36]. It has also been reported that Wnt/β-catenin signaling regulates c-Myc–mediated apoptosis, cytochrome c and caspase activation. Cyclin D1 is a key regulator of cell fate, such as cellular senescence, apoptosis, proliferation and tumorigenesis. The activation of the Wnt/β-catenin pathway due to the loss of its antagonists is associated with gene promoter hypermethylation, which may be involved in the pathogenesis and prognosis of leukemia [37].

RT-PCR, western blot, and IHC staining were used to demonstrate the changes involved in the Wnt/β-catenin pathway. β-catenin and downstream genes, c-Myc and cyclinD1, were downregulated in AML cells both in vivo and in vitro when treated with the sequential combination of DAC and IDA compared with DAC and IDA alone. Moreover, Wnt antagonists SFRP1, HDPR1 and DKK3 were methylated in three AML cell lines. Treatment with DAC allows for the re-expression of Wnt antagonists. However, DAC followed by IDA induces even further increased expression of Wnt antagonists compared to DAC alone. It is also clear that DAC upregulates the expression of various tumor inhibitors by way of demethylation of the gene promoter, resulting in inhibition of cell proliferation [38]. In this study, we found few changes in Wnt antagonist expression levels in the AML cells treated with IDA alone. However, combined treatment resulted in a significant increase in expression levels of these antagonists. This indicates that upregulation of Wnt antagonists and downregulation of Wnt downstream genes enhances an anti-leukemia effect and may partially explain the mechanism behind the combined effect of DAC and IDA.

## Conclusion

We concluded that DAC sequentially combined with IDA has a synergistic anti-leukemia effect in vitro and significant effect on tumor volume reduction and apoptosis induction in a xenograft mouse model. The up-regulation of Wnt antagonists and subsequent inhibition of Wnt/β-catenin signaling with the two drugs in combination may explain this effect. These findings suggest clinical potential in sequential administration of DAC and IDA in the treatment of myeloid leukemia and high-risk MDS.

## Competing interests

The authors declare that they have no competing interests.

## Authors’ contributions

HYT, KFL, and CM participated in the design of the study, data acquisition and analysis as well as drafting the manuscript. KFL, CM, CH, and ZGR were responsible for the laboratory assay and troubleshooting. KFL, CM and ZGR participated in data acquisition, analysis, and interpretation. JJ, ZPZ and JCV conceived of the study, and participated in its design and coordination and helped to draft the manuscript. All authors read and approved the final manuscript.

## Supplementary Material

Additional file 1: Table S1 Three different ways (groups) of combining DAC and anti-leukemia drugs. Click here for file

Additional file 2: Table S3 CI50 of each combination treatment in cell lines and cells from AML patients. Click here for file

Additional file 3: Table S2 IC50 values of each compounds used in this study in human leukemia cells. Click here for file
