# Supplementary material for: Sequential combination of decitabine and idarubicin synergistically enhances anti-leukemia effect followed by demethylating Wnt pathway inhibitor promoters and downregulating Wnt pathway nuclear target
Source: J Transl Med. 2014 Jun 12;12:167. doi: 10.1186/1479-5876-12-167 (PMC4082426; doi:10.1186/1479-5876-12-167)
Supplement: Additional file 1: Table S1 — Three different ways (groups) of combining DAC and anti-leukemia drugs. [file 1479-5876-12-167-S1.doc]

Table S1. Three different ways (groups) of combining DAC and anti-leukemia drugs

|  | *Treatment* | | | |
| --- | --- | --- | --- | --- |
| *Groups* | *0 h* | *24 h* | *48 h* | *72 h* |
| Group1 | DAC+X | - | - | test |
| Group2 | DAC | - | X | test |
| Group3 | DAC | DAC | X | test |

X represent HHT, ACLA, THAL, DNR, IDA respectively. The IC50 value of DAC was 1.06±0.11umol/L in group 1 and 2, 0.78±0.05umol/L in group 3; HHT was 2.74±0.19ng/ml in group 1, 3.66±0.23ng/ml in group 2 and 3; ACLA was 0.23±0.01 ug/ml in group 1, 0.37±0.02ug/ml in group 2 and 3; THAL was 3.76±0.16ug/ml in group 1, 4.31±0.13ug/ml in group 2 and 3; DNR was 0.06±0.01umol/L in group 1, 0.13±0.01umol/L in group 2 and 3; IDA was 71.4±2.31nmol/L in group 1, 100.22±3.89nmol/L in group 2 and 3. In all combination groups, the timing for the IC50 of DAC was 72h. The timing for the IC50 of the other drugs (HHT, THAL, ACR, DNR, and IDA) was 72h in combination group 1, 24h in group 2 and 3.
