# Supplementary material for: Sequential combination of decitabine and idarubicin synergistically enhances anti-leukemia effect followed by demethylating Wnt pathway inhibitor promoters and downregulating Wnt pathway nuclear target
Source: J Transl Med. 2014 Jun 12;12:167. doi: 10.1186/1479-5876-12-167 (PMC4082426; doi:10.1186/1479-5876-12-167)
Supplement: Additional file 2: Table S3 — CI50 of each combination treatment in cell lines and cells from AML patients. [file 1479-5876-12-167-S2.doc]

**Table S3.** CI50 of each combination treatment in cell lines and cells from AML patients

| *Cells* | *Treatment* | *CI 50* | *Dose of individual drugs* |
| --- | --- | --- | --- |
| U937 | DAC+HHT 1 | 0.84±0.01 | DAC: 0.71umol/L/HHT: 1.12ng/ml |
| U937 | DAC+HHT 2 | 1.57±0.04 | DAC: 0.86umol/L/HHT: 2.57 ng/ml |
| U937 | DAC+HHT 3 | 1.23±0.08 | DAC: 0.66umol/L/HHT: 1.59 ng/ml |
| U937 | DAC+THAL 1 | 1.41±0.08 | DAC: 0.60umol/L/THAL: 2.02ug/ml |
| U937 | DAC+THAL 2 | 0.96±0.03 | DAC: 0.53umol/L/THAL: 2.32ug/ml |
| U937 | DAC+THAL 3 | 1.07±0.01 | DAC: 0.55umol/L/THAL: 2.57ug/ml |
| U937 | DAC+ACLA 1 | 1.35±0.08 | DAC: 0.40umol/L/ACLA: 0.18ug/ml |
| U937 | DAC+ACLA 2 | 1.50±0.09 | DAC: 0.47umol/L/ACLA: 0.20ug/ml |
| U937 | DAC+ACLA 3 | 3.91±0.05 | DAC: 0.55umol/L/ACLA: 0.27ug/ml |
| U937 | DAC+DNR 1 | 1.81±0.01 | DAC:0.44umol/L/DNR: 0.05umol/L |
| U937 | DAC+DNR 2 | 1.21±0.04 | DAC: 0.40umol/L/DNR: 0.08umol/L |
| U937 | DAC+DNR 3 | 0.94±0.07 | DAC: 0.56umol/L/DNR: 0.10umol/L |
| U937 | DAC+IDA 1 | 1.04±0.09 | DAC: 0.45umol/L/IDA: 58nmol/L |
| U937 | DAC+IDA 2 | 0.67±0.01 | DAC: 0.34umol/L/IDA: 65nmol/L |
| U937 | DAC+IDA 3 | 0.44±0.01 | DAC: 0.28umol/L/IDA:53nmol/L |
| HEL | DAC+IDA 3 | 0.53±0.04 | DAC: 0.03umol/L/IDA: 46nmol/L |
| SKM-1 | DAC+IDA 3 | 0.66±0.07 | DAC: 4.12umol/L/IDA: 9nmol/L |
| Patient 1 | DAC+IDA 3 | 0.46±0.03 | DAC: 3.90umol/L/IDA: 105nmol/L |
| Patient 2 | DAC+IDA 3 | 0.48±0.05 | DAC: 2.28umol/L/IDA: 127nmol/L |
| Patient 3 | DAC+IDA 3 | 0.32±0.01 | DAC: 3.20umol/L/IDA: 99nmol/L |
