# Supplementary material for: Sequential combination of decitabine and idarubicin synergistically enhances anti-leukemia effect followed by demethylating Wnt pathway inhibitor promoters and downregulating Wnt pathway nuclear target
Source: J Transl Med. 2014 Jun 12;12:167. doi: 10.1186/1479-5876-12-167 (PMC4082426; doi:10.1186/1479-5876-12-167)
Supplement: Additional file 3: Table S2 — IC50 values of each compounds used in this study in human leukemia cells. [file 1479-5876-12-167-S3.doc]

**Table S2. IC50 values of each compounds used in this study in human leukemia cell lines**

|  | *cells* | | | |
| --- | --- | --- | --- | --- |
| *IC50* | *U937* | *HEL* | *SKM-1* | *Cells from AML patient* |
| DAC(umol/L) | 0.78±0.05 | 0.05±0.01 | 9.68±0.27 | 6.75±0.35 |
| IDA(nmol/L) | 100.22±3.89 | 90.88±1.15 | 11.71±0.18 | 163.444±2.16 |

The timing for IC50 of DAC was 72h for U937, HEL and cells from patients, 96h for SKM-1. The timing for IC50 of IDA was 24h for the three cell lines and cells from patients.
